# From Petechiae to Intracerebral Hemorrhage: A Rare Progression of Pediatric Idiopathic Thrombocytopenia

**DOI:** 10.7759/cureus.80818

**Published:** 2025-03-19

**Authors:** Nirupam Nadella, Satwik Kuppili, Rhythm L Shukla, Korvi N Kumar, Adil M Siddiqui, Madhukar Madineni

**Affiliations:** 1 Department of General Medicine, Dr. D.Y. Patil Medical College Hospital and Research Centre, Dr. D.Y. Patil Vidyapeeth (Deemed to be University), Pune, IND; 2 Department of General Medicine, Konaseema Institute of Medical Sciences, Amalapuram, IND

**Keywords:** corticosteroid therapy in itp, glasgow coma scale (gcs) in itp, idiopathic thrombocytopenic purpura (itp), intracerebral hemorrhage, purpura and ecchymosis

## Abstract

A four-year-old boy presented with a four-week history of hematuria, melena, and ecchymoses on his forehead, arm, and back, as well as upper respiratory symptoms. The physical examination revealed diffuse purpura on the lips, pallor, and several hyperpigmented spots. Laboratory results showed microcytic hypochromic anemia, neutropenia, lymphocytosis, and severe thrombocytopenia. A peripheral smear revealed giant platelets and a bone marrow biopsy revealed enhanced megakaryocytes with aberrant maturation. Despite the initiation of prednisolone medication, the patient experienced generalized tonic-clonic seizures and diminished consciousness (Glasgow Coma Scale: 4/15). Neuroimaging revealed a left-sided intracerebral hemorrhage with a midline shift, requiring an immediate decompressive craniotomy. Early detection and rapid interdisciplinary management are critical for improving outcomes in these life-threatening conditions.

## Introduction

Thrombocytopenia is a platelet count of <150 × 10^9^ /L. Low platelets may result from reduced bone marrow production, increased destruction in the circulation (due to coagulopathic consumption, auto-antibodies, vasculopathy, or inflammation), hemodilution, or splenic sequestration [1]. Idiopathic thrombocytopenic purpura (ITP) is one of the most common causes of symptomatic thrombocytopenia in children [2,3], with an annual incidence of 1 to 6.4 cases per 100,000 children [2,4]. Hematoma, petechiae, mucous membrane bleeding, and other hemorrhagic manifestations of thrombocytopenia are the most common clinical symptoms [5,6].

## Case presentation

**Figure 1:**
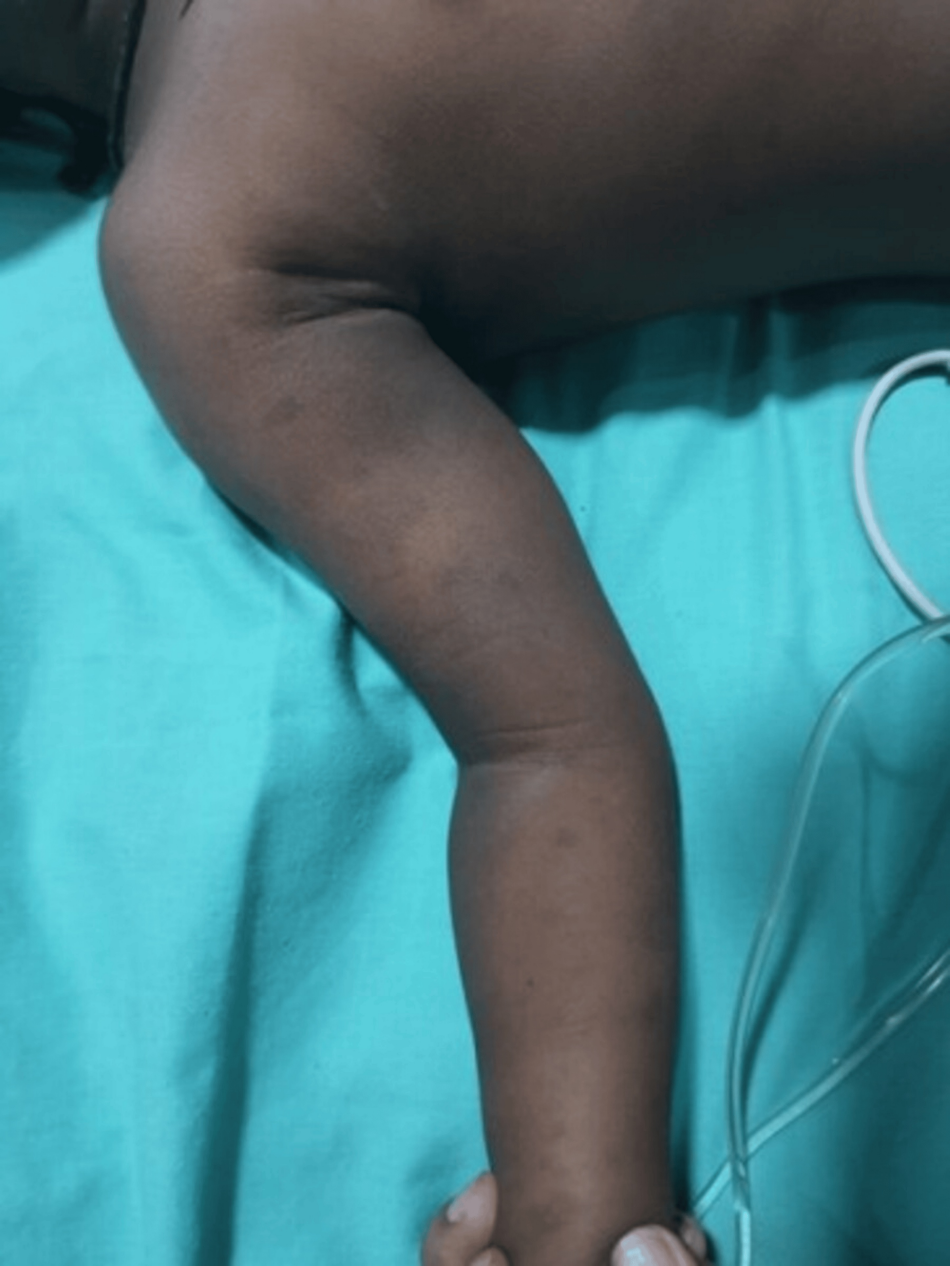
Multiple Hyperpigmented Patches

**Figure 2:**
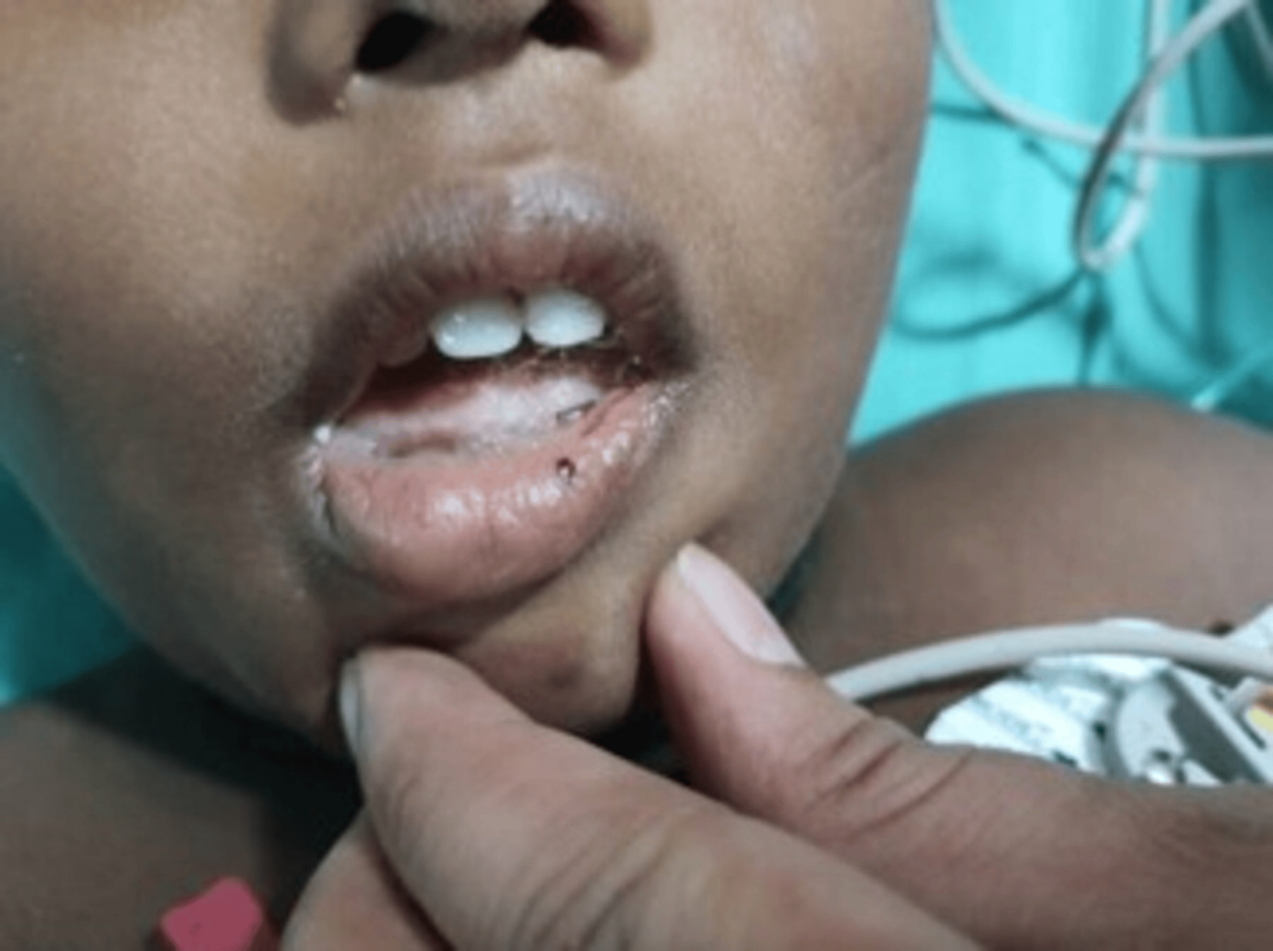
Purpura on the Lips

**Figure 3:**
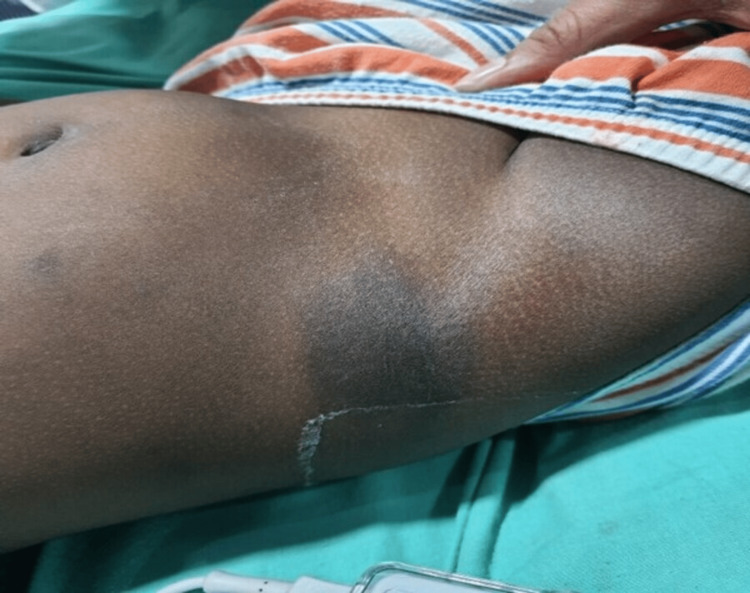
Hyperpigmented Patches on the Thigh

A four-year-old male child presented with a four-week history of red-colored micturition and tarry stools. There was a history of recent cough and cold and black patches over the forehead, arm, and back, as shown in Figure 1, but there was no history of blood transfusion, jaundice, or similar complaints in the past. He had not been taking any medications that could precipitate ITP. On examination, he was afebrile with a blood pressure of 100/60mmHg. There was diffuse purpura on the lips, as shown in Figure 2, and ecchymosis but no rash. Pallor was present, and there were no retinal hemorrhages, gum bleeding, splenomegaly, or lymphadenopathy. Four hyperpigmented patches were over the forehead (4cm×3cm), left pinna (2cm×2cm), inner aspect of the right arm (4cm×5cm), and right lower flank just above the thigh (8cm×8cm), as shown in Figure 3.

**Figure 4:**
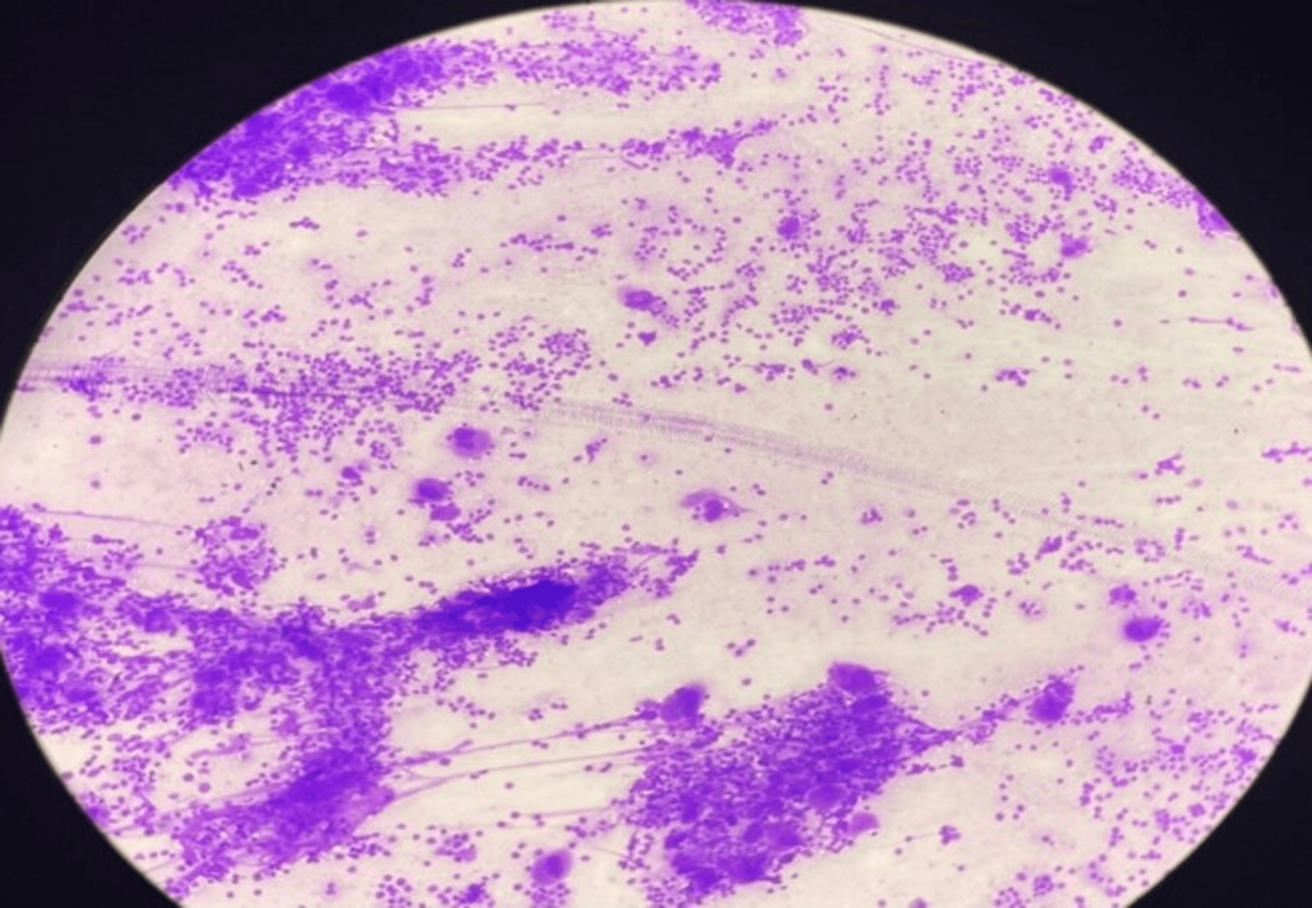
Bone Marrow Aspirate and Biopsy Revealing Increased Megakaryocytes with Dysplastic Maturation

Laboratory investigations revealed microcytic hypochromic anemia with neutropenia, lymphocytosis, and thrombocytopenia and a hemoglobin of 8.2 g/dl (reference value: >11 g/dL), white-blood count of 9100/ cumm (reference range: 5,000 to 15,000 per cumm), platelet count of 0.42 lakhs/cumm, and a reticulocyte count of 3.5% (reference range: 0.5% to 2.0%). Peripheral smear shows giant platelets. The prothrombin time, partial thromboplastin time, thrombin time, fibrinogen, fibrin monomers, and fibrin split products were normal. Serum ferritin and serum electrolytes were normal. An electroencephalogram, gallium scan of the abdomen, and chest X-ray were normal. The bone marrow aspirate and biopsy showed increased megakaryocytes with abnormal maturation, as shown in Figure 4. Platelet antibody levels were not performed because of the profound thrombocytopenia. The workup for the etiology of his thrombocytopenia, including auto-immune and microbiological serology, was entirely negative, and a diagnosis of acute ITP was made. Syrup. Phenylephrine-5mg + Chlorpheniramine-2mg 4ml/TID, Tab. Lansoprazole-1/2 tab/OD, Tab. Folic Acid-150µg/day and Syrup. Zincovit-5ml/OD were initiated. ITP validation led to the start of Tab. Prednisolone-10mg-BD.

**Figure 5:**
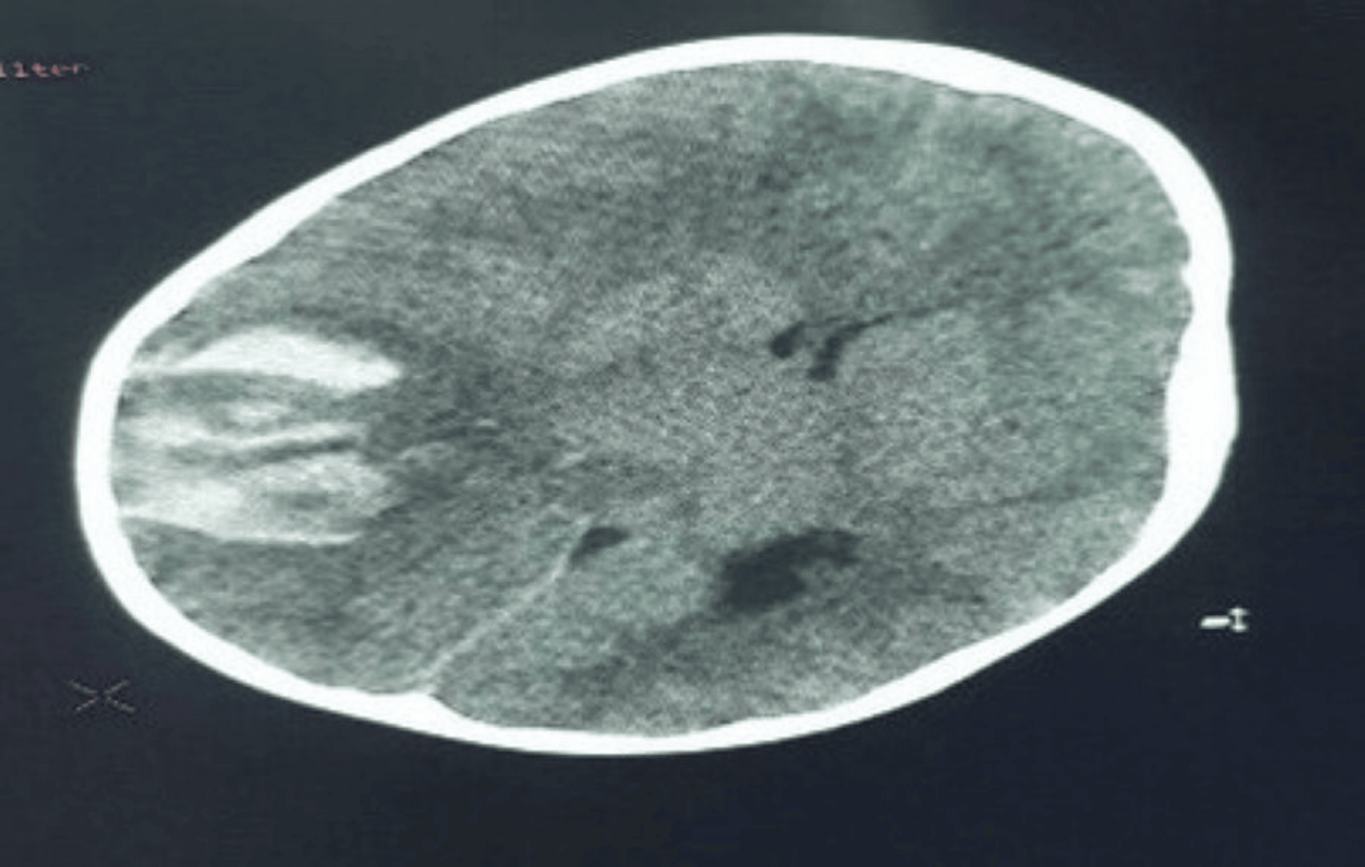
Non-contrast Head CT Showing Left-Sided Intracerebral Hemorrhage with a Midline Shift

The child became drowsy and had generalized tonic-clonic seizures, with a Glasgow Coma Scale of 4/15. The pupils were dilated and not reactive to light. Treatment of status epilepticus started, and the child had decorticate rigidity. A non-contrast head CT was done, showing left-side intracerebral hemorrhage with a midline shift (Figure 5). The patient was referred to neurosurgery, where the neurosurgeon planned for a decompressive craniotomy.

## Discussion

The differential diagnosis for the case could include drug-induced immune thrombocytopenia, hemolytic uremic syndrome, Von Willebrand's disease, acute leukemia, and ITP. Clinical features like ecchymoses and hyperpigmented patches, the absence of splenomegaly, laboratory investigations revealing microcytic hypochromic anemia, and a decrease in platelet count with giant megakaryocytes led to the diagnosis of acute ITP with microcytic hypochromic anemia.

Idiopathic thrombocytopenia could be of two types, the acute and the chronic type, the acute type being more common in children from 2 to 6 years of age [7]. Most cases of Idiopathic thrombocytopenia are preceded by infections with a latent period of around two weeks before the onset of ecchymoses. Most infections are upper respiratory tract infections, particularly viral infections [8]. In a study by Lusher and Zuelzer on 152 children with thrombocytopenic purpura, 83.6% of individuals showed the presence of antecedent infections before purpura [8]. The most common presenting symptoms would be bleeding gums, bruises, petechiae, and nose bleeds. Cerebral hemorrhage is rare, but it is the most serious complication of thrombocytopenic purpura. Anemia is commonly associated with blood loss through nosebleeds and bleeding gums [8].

The laboratory findings show microcytic hypochromic anemia and thrombocytopenia, which is followed by rare cases of severe iron deficiency anemia. However, the expected serum ferritin levels in the biochemical findings make it back to being idiopathic. Anemia and thrombocytopenia might indicate severe bone marrow dysfunction [9]. Cerebral hemorrhage, as in this case, is very rare and indicates the severity of the disease. In a study conducted on 278 patients with thrombocytopenic purpura, only 19 (6.83%) progressed to cerebral hemorrhage. Out of the 19, 17 hemorrhages occurred in the first month of the disease and are associated with various spontaneous bleedings all over the body [8]. 

Thrombocytopenic purpura is generally treated with corticosteroids, intravenous immunoglobulins as the first line of therapy, and immunosuppressants as the second line. In progressed stages, surgical intervention may be required, and a splenectomy has to be done [10]. However, the usage of corticosteroids in cases of ITP has been controversial. It is suggested that steroid usage in the early stages might cause the rise of platelets in some patients and control hemorrhage in other patients whose platelet count remains low [8]. Schulman et al. suggested the infusion of fresh plasma to patients with idiopathic thrombocytopenia at a dose of 30ml per kg body weight [11], and this is currently being followed by many practitioners [8].

## Conclusions

This case highlights the severity and unpredictable progression of acute ITP, particularly when complicated by intracerebral hemorrhage. While ITP is often a self-limiting condition in children, this case underscores the need for close monitoring and timely intervention, especially in patients presenting with severe thrombocytopenia and signs of systemic involvement. The rare occurrence of cerebral hemorrhage in ITP serves as a reminder that early detection, aggressive management, and multidisciplinary coordination are crucial to improving patient outcomes. Future research and clinical vigilance are necessary to refine treatment protocols and identify patients at risk for such severe complications.
